# Physiological oxygen measurements *in vitro-*Schrödinger’s cat in 3D cell biology

**DOI:** 10.3389/fbioe.2023.1218957

**Published:** 2023-10-11

**Authors:** Eric Gottwald, Christoph Grün, Cordula Nies, Gregor Liebsch

**Affiliations:** ^1^ Institute of Functional Interfaces, Karlsruhe Institute of Technology, Karlsruhe, Germany; ^2^ PreSens Precision Sensing GmbH, Regensburg, Germany

**Keywords:** physiological oxygen concentration, *in vitro*, 3D cell culture, organoid, microcavity sensor arrays, optical O_2_ measurement

## Abstract

After the development of 3D cell culture methods in the middle of the last century and the plethora of data generated with this culture configuration up to date, it could be shown that a three-dimensional arrangement of cells in most of the cases leads to a more physiological behavior of the generated tissue. However, a major determinant for an organotypic function, namely, the dissolved oxygen concentration in the used *in vitro*-system, has been neglected in most of the studies. This is due to the fact that the oxygen measurement in the beginning was simply not feasible and, if so, disturbed the measurement and/or the *in vitro*-system itself. This is especially true for the meanwhile more widespread use of 3D culture systems. Therefore, the tissues analyzed by these techniques can be considered as the Schrödinger’s cat in 3D cell biology. In this perspective paper we will outline how the measurement and, moreover, the regulation of the dissolved oxygen concentration *in vitro*-3D culture systems could be established at all and how it may be possible to determine the oxygen concentration in organoid cultures and the respiratory capacity via mito stress tests, especially in spheroids in the size range of a few hundred micrometers, under physiological culture conditions, without disturbances or stress induction in the system and in a high-throughput fashion. By this, such systems will help to more efficiently translate tissue engineering approaches into new *in vitro*-platforms for fundamental and applied research as well as preclinical safety testing and clinical applications.

## 1 Introduction

Since the introduction of 3D cell culture techniques by (Holtfreter et al., 1944; Moscona et al., 1957) in the middle of the last century, it has been shown that three-dimensional *in vitro*-approaches are superior to their two-dimensional counterparts in many respects. This superiority is characterized by changes in key cellular parameters that influence the organotypic behavior such as morphology (Puschmann et al., 2013; Li et al., 2017), ability to establish cell-cell and cell-matrix contacts (Duval et al., 2017; Kapałczyńska et al., 2018; Yamada et al., 2022), proliferation behavior (Kapałczyńska et al., 2018; Souza et al., 2018), metabolism (Tidwell et al., 2022; Rybkowska et al., 2023), differentiation capacity and differentiation status (Altmann et al., 2008; Li et al., 2017; Gaut et al., 2020; Mora-Roldan et al., 2021), mechanotransduction (Chhetri et al., 2021; Man et al., 2022), and drug sensitivity (Stock et al., 2016; Bell et al., 2018). Consequently, this knowledge has led to the development of tissue engineering approaches that better recapitulate the *in vivo*-situation and therefore deliver much more reliable data with regard to transferability to the human situation. One aspect that considerably contributed to that was the development of organoid technology. Organoids can be generated from primary cells as well as embryonal or induced pluripotent stem cells. Once the latter have been appropriately differentiated, they display an unprecedented level of physiomimetic function. Meanwhile, organoid technology is able to generate tissues from all three germ layers with skin (Lee et al., 2020) and brain organoids as derivatives of the ectoderm (Bagley et al., 2017), with kidney (Morizane et al., 2015), blood vessels (Wimmer et al., 2019) and heart (Drakhlis et al., 2021) as mesodermal derivatives, and liver (Hu et al., 2018), small intestine (Sato et al., 2009), and colon (Sugimoto et al., 2018) as examples of endodermal derivatives.

**FIGURE 1:**
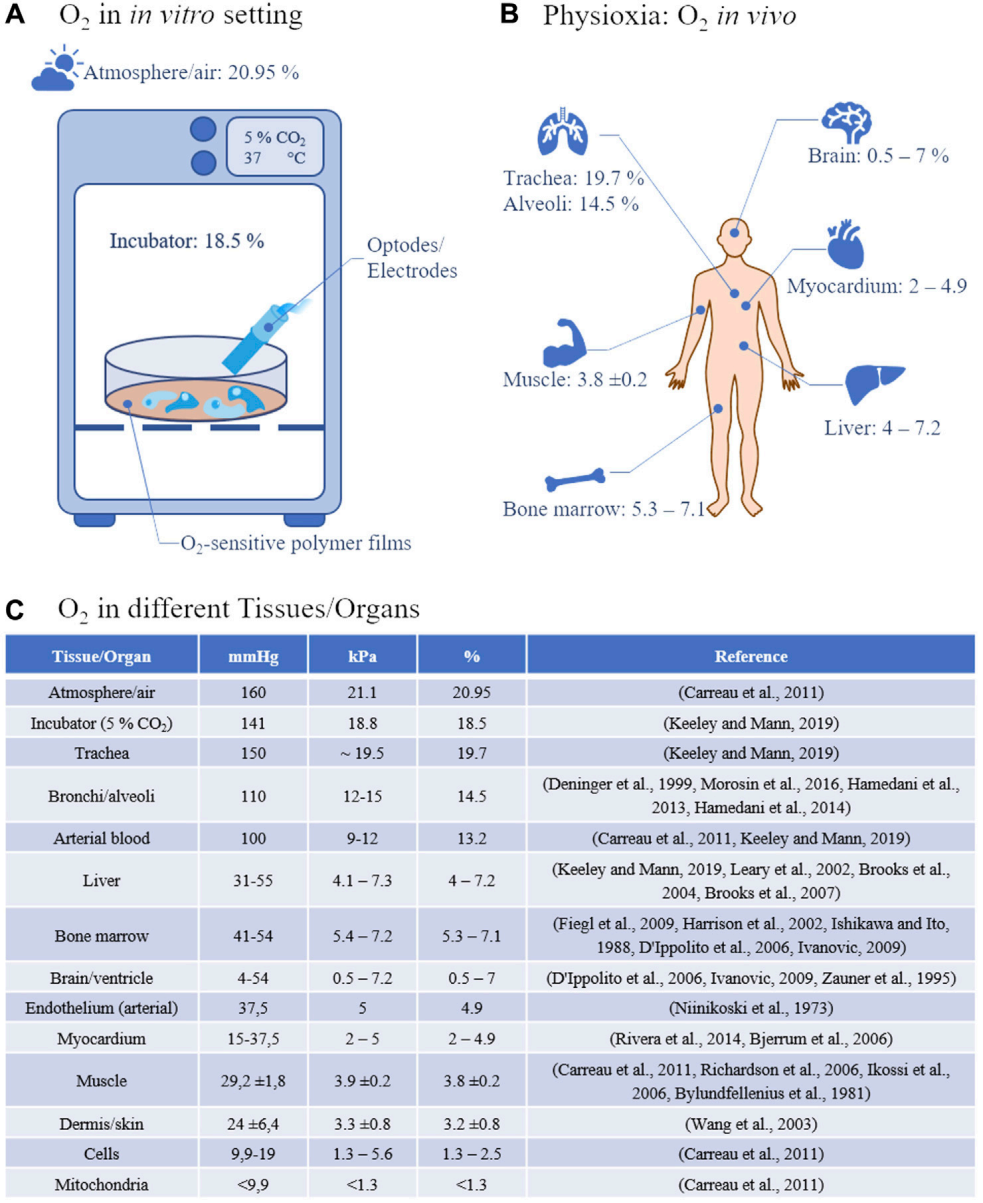
**(A)** Oxygen concentration and measurement *in vitro*: Under standard incubator conditions, 21% oxygen translates into 18.5% oxygen at 37°C, 5% CO_2_, and increased humidity. Measurements in 2D cell cultures are currently carried out either classically via optodes or electrodes or via oxygen-sensitive polymer films, e.g., on the bottom of the cell culture vessels. **(B)** Physioxia describes tissue-specific oxygen concentrations *in vivo*. The variations between the single tissues are enormous from, e.g., almost 20% in the trachea to less than 1% in the brain. **(C)** Typical air and tissue oxygen partial pressures in the most common measurement units.

But as obvious from the above-mentioned list of parameters and tissue engineering approaches that have extensively been characterized, only a few studies dealt with the oxygen concentration and its regulation in the used culture system although it has been shown as early as 1972 that neoplastic and non-neoplastic fetal tissues from mouse and rat have an equal or better plating efficiency in 1%–3% oxygen compared to 20% (Richter et al., 1972). Lewis lung tumor and B16 melanoma cells taken directly from the mouse showed an *in vivo*-like behavior with regard to survival after irradiation when cultured under 5% oxygen in agar gels for 8–15 days (Courtenay, 1976). It was also shown that colony formation of hematopoietic cells resulted in increased colony numbers as well as larger colonies when cultivated under 5% oxygen in contrast to 18% (Bradley et al., 1978). Typically, animal cells are reported to have been cultured at 37°C and under “ambient atmosphere” which refers to an oxygen concentration of 20.9% in air. First of all, this concentration is typically not reached in conventional incubator atmospheres equipped with a CO_2_ supplementation and its higher humidity, leading to a typical concentration of only 18.5% oxygen in the incubation chamber (Keeley and Mann, 2019) (Figure 1A). Secondly, even the 18.5% oxygen concentration is not reached, apart from the trachea, in any human or animal tissue but still, cell culture is routinely performed at this concentration (Figures 1B, C).

Moreover, this is oftentimes termed “normoxia” which, from the cell’s/tissue’s perspective, is a hyperoxia. This hyperoxia leads to oxygen stress on a cellular level with the generation of reactive oxygen species (ROS) and their influence on, e.g., the NrF2-, NF-κB- and MAPK-signaling cascade (Alva et al., 2022). Although small concentrations of reactive oxygen species are used in cellular signaling (Droge, 2002) and in the short term, can be compensated for due to a number of defense mechanisms that have evolved over time, in the long run, too high ROS concentrations are implicated in being involved in the generation of several diseases such as cancer (Jelic et al., 2021), male infertility (Bisht et al., 2017), type 2 diabetes mellitus (Bigagli and Lodovici, 2019), heart failure (van der Pol et al., 2019), and aging (Liguori et al., 2018), among others. On the other hand, a hypoxia is believed to play a major role in many diseases as well (Semenza, 2014). This leads us to the necessity of defining a tissue specific normoxia which might be more appropriately be termed as physioxia (Carreau et al., 2011). When looking into the literature, this physioxia, as data are available at all, is measured either as a concentration in µM, or as a physiologically more relevant partial pressure, reported in kPa or mmHg whereas oxygen in *in vitro*-systems is often determined as the dissolved oxygen concentration given in % of the respective medium with 1% oxygen corresponding to 1.02 kPa or 7.65 mmHg. To adjust oxygen to physiological levels *in vitro* it is imperative to measure physioxia in body fluids and living tissues. For this, several measurement techniques have been developed of which the Clark-type electrodes (Clark et al., 1953) and the Whalen-type electrodes (Whalen et al., 1973) were used to determine the pO_2_ in body liquids and tissues, respectively. However, due to their measurement principle of reducing O_2_ on a polarized electrode that consumes oxygen and which is becoming more difficult the smaller the sample volume is, renders this technique less accurate when measuring low pO_2_ levels. Moreover, the piercing of the tissue with one or more electrodes, to be able to measure more representative O_2_ levels, may damage the tissue leading to altered O_2_ values (Jamieson and van den Brenk, 1965). This and the fact of being hardly able to control the post-diffusion into the penetration channel is making the tissue with this kind of measurement technique to a kind of Schrödinger’s cat in cell biology. Alternative techniques such as hemoglobin/myoglobin spectroscopy which measures changes in the absorption characteristics upon binding O_2_ can be converted into an approximate pO_2_ although this conversion is subject to changes in local pH/pCO_2_ and temperature and hence should be used with caution (Keeley and Mann, 2019). Moreover, hemoglobin/myoglobin is rarely used in *in vitro*-systems. An interesting clinical measuring technique is the so-called dynamic contrast-enhanced near-infrared spectroscopy (NIRS) (Davies et al., 2015) using indocyanine green (ICG) (Forcione et al., 2020). With the help of this technique it could be shown that there is a mismatch between the tissue partial oxygen pressure and tissue respiration (Forcione et al., 2021). This is an extremely important finding since it shows that even in case of a sufficient oxygen partial pressure, the parenchymal tissue respiration might be impaired. Therefore, it is important to look at both aspects in *in vitro-systems* as well. More sophisticated techniques to measure tissue oxygen like, e.g., magnetic, paramagnetic, and electron spin resonance techniques require expensive instrumentation, dedicated and trained personnel, as well as considerably more space which is why these techniques are not very common. In addition, the spatial resolution here is more useful for mapping, e.g., rodents rather than very small organoids, which is of course counterproductive if one wants to reduce animal experiments. With the development of phosphorescent dyes which react with the so-called dynamic quenching effect upon contact with O_2_, a new method for the detection of tissue oxygen concentrations was possible (Rumsey et al., 1988). The effect is based on the collision of an O_2_ molecule with a luminophore in its lowest excited state, resulting in a radiationless deactivation and return to the ground state both of which can be measured. As a result, luminescence intensity (I) and lifetime (τ) are reduced in the presence of O_2_. These luminophore reporter dyes, among others, comprise Pt (II)- and Pd(II)-porphyrine complexes that are suitable for oxygen measurements in the range of 0–200 µM due to their quenching effects (Dmitriev and Papkovsky, 2012). Moreover, the reporter dyes can be formulated into different formats by immobilizing them in special polymers that allow oxygen diffusion but prevent the dyes from bleeding into the measurement medium so that extra- (Dunphy et al., 2002) and intracellular (Neugebauer et al., 2008) measurements can be performed. By this, very high spatial resolutions are possible (Blancke Soares et al., 2021). Meanwhile, there is a wide variety of fluorescence optical oxygen sensors available (Wang and Wolfbeis, 2014).

In general, the fluorescence optical sensor technique has led into the development of sophisticated instruments with which mitochondrial respiration and glycolytic activity of monolayers can be measured in real-time metabolic assays (Agilent Technologies, Incyton) that have led to so-called mito stress test and mito complex assays. Although this type of assay is a very powerful technique to test the cellular reactions upon, e.g., drug administration, the system is neither able to perform these measurements in 3D cultures nor can they be performed under physiological conditions. Due to the used measurement technique that requires the lowering of the measuring device into the well and thereby blocking atmospheric exchange, a severe hypoxia is generated leading to experiment durations of only 30–60 min. However, one of the greatest advantages of the luminescence approach is the fact that the required reporter molecules can be incorporated into polymer matrices like polystyrene, with or without covalent linkage to the polymer backbone (Babilas et al., 2005). Anyway, up to now this was only possible in two-dimensional *in vitro*-cultures.

What significantly adds up to the non-physiological *in vitro*-culture conditions is the lack of a flow. Most of the experimental (oxygen) data available have been generated in static culture systems which do not provide a physiological environment. This changed with the introduction of microbioreactors that not only miniaturized the systems, thereby being able to parallelize experiments, but moreover were able to incorporate an active flow. Beginning with rather simple setups, like the PDMS (polydimethylsiloxane) perfusion microbioreactor for the 3D culture of chondrocytes (Wu et al., 2006), this area of research developed into the major driver for the advancement of organotypic culture systems, that is, currently represented in many types of single- or multi-organ-, or human/body-on-a-chip (OOAC) systems. But although capabilities for, e.g., mechanical or electrical stimulation can be incorporated, only a few systems are capable of on-chip analysis of the metabolic and physiological behavior of the tissue and are analyzed by classical off-chip technologies such as PCR, enzyme-linked immunosorbent assays (ELISA), and mass spectrometry (MS) (Li and Tian, 2018). Again, a major driver for the incorporation of sensors that are capable of characterizing online and in real-time the status of the culture in OOAC systems, was miniaturization which was used in lab-on-a-chip systems for the analysis of smaller volumes in shorter times. Basic sensing principles comprise electrical, electrochemical, and optical techniques. Within the electrical sensor family, trans-endothelial/endothelial resistance (TEER) electrodes (Odijk et al., 2015) have become relatively common to characterize the barrier function of cells. For the characterization of electrically active cells, the extracellular field potential measurement technique, realized in multielectrode arrays (MEA), can be employed (Koutsouras et al., 2017). Among electrochemical sensors, potentiometric and amperometric sensors can be distinguished. Both are making use of the transformation of an electrochemical interaction between an analyte and an electrode (Fuchs et al., 2021) leading to a read-out, that is, either a voltage difference or a current flow between two electrodes. Common analytes for this measurement technique are oxygen and pH with the disadvantages in oxygen measurements as discussed earlier. Optical sensors are based on detecting changes in an optical property, such as luminescence, absorption, refractive index, or scattering (Fuchs et al., 2021). Of these, photoluminescence is a very attractive approach for measuring oxygen since it does not interfere with the analyte and the tissue because the required fluorophores (indicator and reference dyes) can be incorporated into a polymer matrix thereby passivating the sensor and separating the indicator dyes from the tissue. With this technique, a sensitivity of 0.067 Pa for oxygen can be achieved (Wolfbeis et al., 1986).

## 2 Paving the road for oxygen measurements in 3D cultures

**FIGURE 2:**
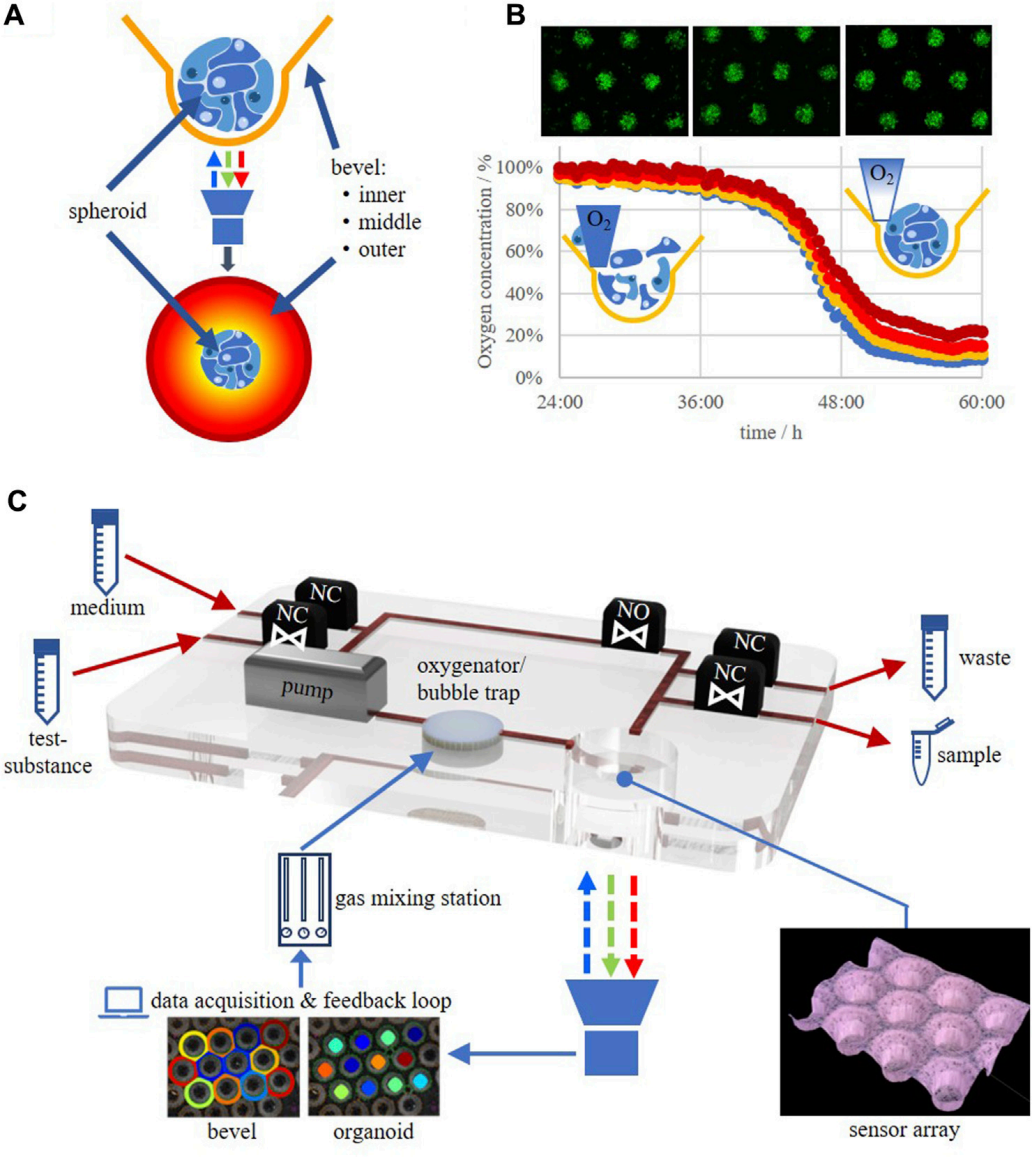
**(A)** Spheroid/organoid in an oxygen-sensitive microcavity. An oxygen gradient is established by realizing an oxygen post-diffusion from the culture medium to the cells mimicking the situation in post-capillary tissues. The dissolved oxygen concentration in the direct microenvironment of the spheroid/organoid as well as oxygen gradients at the upper side of the microcavity can be measured. **(B)** HepG2 cells seeded as a single cell suspension into a sensor array and the corresponding change in oxygen levels in the time frame from 24 to 60 h after seeding. **(C)** Schematic drawing of an oxygen-feedback regulated microbioreactor circulation. NO, valve, normally open; NC, valve, normally closed.

So far, only very limited studies showing oxygen measurements in 3D culture systems are available. One of the approaches used a needle-type sensor equipped with an optical sensor foil which was mounted on computer-aided micromanipulator for *z*-axis oxygen profiling in collagen gels (Wolff et al., 2019). A similar system, capable of increasing the throughput by automation and therefore, to perform microphysiological measurements in real-time over extended cultivation periods, was introduced by Eggert et al. (2021). A phosphorescence lifetime-based system is able to measure the dissolved oxygen concentration even within 3D cellular constructs by incorporating oxygen-sensitive microbeads (Weyand et al., 2015; Wesseler et al., 2022). However, as discussed above, these measurement techniques only deliver spot-like measurement data, may destroy the tissue architecture within the piercing channel with the associated adverse effects on the cells, and are not able to characterize the cells’ metabolism further. Multiplexing is also limited by, e.g., the fiber optic cable management or the system setup. It would therefore be of prime importance to be able to measure oxygen in a multiplex-fashion in 3D culture systems in a real-time, label-free, oxygen consumption-free method in the direct microenvironment of the tissue under physiological conditions. Such a system has recently been introduced by Grün et al. (2023) and was realized by using an oxygen-sensitive polymer film that was subjected to a microthermoforming process resulting in an oxygen-sensitive microcavity array, so-called sensor arrays. The sensor arrays cannot only be used as the system for the generation and culture of the spheroids/organoids themselves (self-assembly of 3D aggregates which show very similar sizes and shapes by simply pipetting the cells into form-exact microcavities) but moreover deliver oxygen data from the direct environment of the aggregates. The system also delivers oxygen gradient profiles due to the integrated bevel on the upper side of the microcavities (Figure 2). This would deliver the necessary information of the tissue supply with oxygen via diffusion which would compare to perfusion of tissues with indocyanine green to characterize tissue integrity (Forcione et al., 2020).

By the help of the sensor array technology it would now be feasible to run, e.g., mito stress tests under physiological conditions, unlike those that are currently performed in 2D cultures which are, furthermore, restricted in time due to the fact that the measurement principle generates a hypoxic atmosphere itself. Now, 3D mito stress tests, for the characterization of the respiratory capacity of 3D cultures/organoids can be performed which was recently demonstrated (Grün et al., 2023). By such techniques it will now be possible to determine the oxygen concentration for individual cell types under physiologically relevant concentrations and/or best physiomimetic function. This could be achieved by, e.g., determining the expression of typical tissue markers on a genetic as well as a protein level and/or enzymatic functions. By this, a database will evolve in which optimal *in vitro* oxygen concentrations are collected, leading to an oxygen footprint for each individual cell type, be it a cell line or a primary cell. Therefore, such systems will help to more efficiently translate tissue engineering approaches into new *in vitro*-platforms for fundamental and applied research as well as preclinical safety testing and clinical applications.

## 3 Discussion

We are just at the beginning of a new era in *in vitro* cell biology in which we will be able to not only culture cells in 3D but moreover in an organotypic way that will resemble the tissue of origin in an unprecedented manner. This will largely be achieved by incorporating oxygen measurement techniques into the culture systems that do not interfere with the culture itself and do not change the culture inherent oxygen concentrations. This being said, it is only a small step to 3D culture systems that use the oxygen measurement data as an input for a feedback-regulated measurement environment in which the cells regulate the optimal oxygen concentration themselves (Figure 2). The application of a regulated oxygen tension via the culture medium into the 3D cell construct mimics the natural supply process in vascularized tissue, or more precisely, the process taking place in the post-capillary tissue zone. This will finally overcome the problems with the so far called normoxia in current *in vitro* cultures which at the same time will generate the need to establish new standards for the cultivation of the different cells currently in use. Associated with this is a critical evaluation of older data because the physioxic data may differ from what has been measured under hyperoxic conditions that formerly have been designated as “normoxic conditions” (18.5% oxygen).

As usual, the technological development will set the pace for an even further advancement of physiomimetic *in vitro* culture systems. Currently, artificial intelligence is finding its way into image analysis to judge the status of the culture, organoid intelligence is used as a means of a genuine biological computing that harnesses brain organoids using scientific and bioengineering advances in an ethically responsible manner (Smirnova et al., 2023), exosomes and liquid crystal biosensors can be used for the early diagnosis of cancer and pathogens (Pani et al., 2023; Shin et al., 2023), short-wave infrared quantum dots have been shown to act as molecular probes for the labeling and detection of single antibodies, growth factors, and nucleic acid molecules in fluorescence microscopy applications (Sarkar et al., 2020), to name just a few. In any case will the new systems be smaller and smarter, will decrease the resource needs thus making the systems economically more attractive, will deliver more reliable results and by this, will dramatically increase the transferability to the *in vivo* situation.

## Data Availability

The original contributions presented in the study are included in the article/Supplementary Material, further inquiries can be directed to the corresponding author.
